# A280 DOES THE PLACEMENT OF A MANOMETRY PROBE WITH ENDOSCOPY AND CONSCIOUS SEDATION EFFECT MEASUREMENTS ON ENSUING HIGH RESOLUTION MANOMETRY? A RETROSPECTIVE COHORT STUDY IN PATIENTS POST-PERORAL ENDOSCOPIC MYOTOMY

**DOI:** 10.1093/jcag/gwac036.280

**Published:** 2023-03-07

**Authors:** N K Sandhu, R Bechara, D M Rodrigues

**Affiliations:** Department of Medicine, Queen's University, Kingston, Canada

## Abstract

**Background:**

High resolution manometry (HRM) is the gold standard for diagnosis of esophageal motility disorders. This is traditionally conducted without sedation, using intranasal intubation with a manometry probe. Inserting the HRM probe immediately after endoscopic examination allows for endoscopic visualization and sedation for probe placement. This method is used commonly used for our patients who are post-peroral endoscopic myotomy (POEM). However, it is unclear whether short acting sedative drugs administered during endoscopy can affect contractility measurements during HRM.

**Purpose:**

The purpose of this study was to determine if conscious sedation with short-acting anaesthetic agents (Midazolam and Fentanyl) influenced HRM readings in patients undergoing evaluation post-perioral endoscopic myotomy (POEM).

**Method:**

We conducted a retrospective cohort study, comparing patients post-POEM who had endoscopy under conscious sedation with placement of the manometry probe versus those without preceeding endoscopy/sedation. Post-POEM patients who had undergone HRM over a 3-year period (2019-2022) were identified from motility lab records. Electronic charts were reviewed for data abstraction, and HRM parameters (resting lower esophageal pressure and integrated relaxation pressure) were determined for both groups and compared using t-tests, and error listed as standard error of the mean.

**Result(s):**

From 2019-2022, 15 patients underwent manometry using a non-endoscopic approach with no sedation and 25 underwent manometry following endoscopy with conscious sedation and placement of the probe during the procedure. In the latter group, HRM occurred within 2 hours of probe placement. The mean doses of fentanyl and midazolam were 115mcg (+/-29.7) and 3.3mg (+/-1.3) respectively. The mean end-expiratory lower esophageal sphincter pressure was 9.8 mmHg (+/- 2.9) in the non-endoscopic group and 26.24mmHg (+/- 5) in the endoscopy group (p <0.02). The median integrated relaxation pressure was 11.2 mmHg (+/-1.2) in the non-endoscopic group and 14.52mmHg (+/- 0.9) in those in the endoscopy group (p <0.04).

**Conclusion(s):**

In conclusion, this small, retrospective cohort study shows that manometry probe placement after endoscopy with conscious sedation may affect esophgeal HRM measurement parameters in the post-POEM patient population. Physicians should consider delaying HRM measurements for >4 hours to maximize clearance of the sedative medications.

**Please acknowledge all funding agencies by checking the applicable boxes below:**

None

**Disclosure of Interest:**

None Declared

